# The role of the pre-supplementary motor area in the control of action

**DOI:** 10.1016/j.neuroimage.2007.03.034

**Published:** 2007

**Authors:** Parashkev Nachev, Henrietta Wydell, Kevin O’Neill, Masud Husain, Christopher Kennard

**Affiliations:** a Department of Clinical Neuroscience, Imperial College London, St Dunstan’s Road, London W6 8RP, UK; b West London Neurosciences Centre, Charing Cross Hospital, London W6 8RF, UK; c Institute of Neurology and Institute of Cognitive Neuroscience, UCL, Alexandra House, 17 Queen Square, London WC1N 3AR, UK

## Abstract

Although regions within the medial frontal cortex are known to be active during voluntary movements their precise role remains unclear. Here we combine functional imaging localisation with psychophysics to demonstrate a strikingly selective contralesional impairment in the ability to inhibit ongoing movement plans in a patient with a rare lesion involving the right pre-supplementary motor area (pre-SMA), but sparing the supplementary motor area (SMA). We find no corresponding delay in simple reaction times, and show that the inhibitory deficit is sensitive to the presence of competition between responses. The findings demonstrate that the pre-SMA plays a critical role in exerting control over voluntary actions in situations of response conflict. We discuss these findings in the context of a unified framework of pre-SMA function, and explore the degree to which extant data on this region can be explained by this function alone.

## Introduction

A region within the medial frontal cortex – seemingly at the interface between pre-frontal and motor systems – has become the focus of great interest in recent years. The supplementary motor complex (SMC) occupies a rostrally ill-defined area of mesial agranular frontal cortex immediately anterior to Brodmann Area 4 (BA 4) (Freund, 1996; Picard and Strick, 1996; Zilles et al., 1996). Within this region, a fractionation into at least two, functionally distinct divisions – the pre-supplementary motor area (pre-SMA) and the more caudal supplementary motor area (SMA) proper (Matsuzaka et al., 1992) – is strongly suggested both on grounds of cytoarchitectonics (Matelli et al., 1991; Vorobiev et al., 1998) and strikingly different cortical and subcortical connectivity (Behrens et al., 2006; Inase et al., 1999; Johansen-Berg et al., 2004; Luppino et al., 1993; Morel et al., 2005; Wang et al., 2005). Unlike its rostral counterpart, the SMA is somatotopically organised (Chainay et al., 2004; He et al., 1995), has direct projections to spinal cord (He et al., 1995; Luppino et al., 1994) and primary motor cortex (Luppino et al., 1993), but receives no pre-frontal inputs (Luppino et al., 1993). By contrast, the pre-SMA has extensive pre-frontal connectivity (Luppino et al., 1993; Wang et al., 2005), a feature shared by the supplementary eye field (SEF) (Tehovnik et al., 2000), a region found at the pre-SMA/SMA border (Grosbras et al., 1999; Yamamoto et al., 2004) with marked activity during eye movements, but functional characteristics not dissimilar from the pre-SMA (Coe et al., 2002; Fujii et al., 2002; Husain et al., 2003; Isoda and Tanji, 2003).

There are good anatomical grounds, then, for the wide range of “higher” functions in which this region – and the pre-SMA in particular – has been implicated: language generation (Binder et al., 1997; Wise et al., 1991), movement recognition and ideation (Grafton et al., 1996; Stephan et al., 1995), maintaining working memory (Pollmann and von Cramon, 2000), establishing visuomotor associations (Sakai et al., 1999), learning and performing movement sequences (Hikosaka et al., 1996; Isoda and Tanji, 2004; Kawashima et al., 1998; Nakamura et al., 1998, 1999; Sakai et al., 1998; Shima and Tanji, 1998, 2000), time perception and discrimination (Coull et al., 2004; Pastor et al., 2006), “internally” guided action (Deiber et al., 1991; Frith et al., 1991; Hadland et al., 2001; Lau et al., 2006), representing action intentions (Lau et al., 2004), conflict resolution or monitoring (Garavan et al., 2003; Nachev et al., 2005; Ullsperger and von Cramon, 2001), and switching between action sets (Kennerley et al., 2004; Matsuzaka and Tanji, 1996; Rushworth et al., 2002; Shima et al., 1996). However, it is possible that the multiplicity of functions these data have been interpreted as indicating is in fact a multiplicity of aspects of a much smaller range of fundamental functions, possibly even just one: the resolution of competition between motor plans so that a unitary action may emerge.

It may be argued that such a process of resolving competition between conflicting motor plans is fundamental to action control (Botvinick et al., 2001). At any point in time, we are faced with a choice of actions, the majority of which are either incompatible or likely to interact with one other. A critical question is therefore how one action is selected in favour of another. Positing a brain area that *chooses* the specific neural circuit required to execute the action leads to an infinite regress: one would have to explain what chooses its choice, and so on *ad infinitum*. Much more plausibly, the action ultimately performed is the outcome of a competition within a space of contingent *condition–action associations* whose neural representations are variously activated by current external stimuli as well as internal biases.

We use the term condition–action associations rather than the more conventionally used ‘stimulus–response (S–R) associations’ for a specific reason. S–R associations refer to the links between an action and a particular stimulus in the environment, e.g. stop at a red light. In experimental situations, animals are often required to learn, by trial and error, the appropriate response for a particular arbitrary stimulus, e.g. move hand to top right when there is a red circle but to bottom left when there is a blue square. Positive or negative feedback following the animal’s response to each of these stimuli leads to the development of strong S–R associations. These associations need not only involve external stimuli, but also an internal state or combinations of many external stimuli and internal states, making the term condition–action associations more appropriate.

Critically, in a given context and at some level, a multitude of neural representations *must* be co-activated *automatically* by external stimuli, internal biases, and conditional associations between the two. There can be no homunculus determining which representation should be activated and which should not. Without a mechanism to resolve this competition, coherent action would fail to emerge because of interference from actions incompatible with the one being performed. Importantly, such a mechanism does not do any *selection* – as the competition itself is the selection – but merely reinforces the winners and inhibits the losers of the competition.

If such a mechanism exists, one would expect its activity to be sensitive to at least three things. First, and most obviously, it will depend on the degree of incompatibility between the possible actions whose representations are activated (degree of conflict). Second, it will depend on how greatly one action plan is biased over the others (degree of bias): where there is a pre-existing bias in favour of one action, competition would be expected to be less. Third, it will vary with the number of condition–action associations concurrently activated (condition–action dimensionality) in a context-specific fashion depending on attention and other processes that may introduce bias towards specific behavioural sets.

This framework allows us to make specific and testable predictions. If the pre-SMA is indeed involved in this conflict-resolving process, we would expect its inactivation to lead to a deficit in the ability to select one movement over another when two movement plans are concurrently activated. Using a manual paradigm designed to expose such a deficit (Husain et al., 2003; Logan et al., 1984), we therefore tested a rare patient with a unilateral lesion involving the whole of the right pre-SMA, but – as we demonstrate by functional imaging – entirely sparing the SMA proper, allowing for a dissociation between the two areas. We also show that the patient’s lesion incorporates a region of the pre-SMA responsive to conflict identified by functional imaging of the same basic paradigm (Nachev et al., 2005).

## Patient and methods

Patient AG, a previously fit right-handed woman, presented aged 52 following two generalised seizures. Neurological examination was unremarkable. Structural MRI revealed a right dorsomedial frontal lesion whose margins functional MRI confirmed to be anteromedial to hand primary motor cortex. A tumour (grade 2 oligoastrocytoma) was surgically completely removed in conjunction with intra-operative electrical stimulation ensuring that none of the areas designated for resection could elicit a motor response. Immediately after the operation, there was motor neglect of the left upper limb which resolved spontaneously. The experiments described here were performed 22 months after surgery when the patient was entirely asymptomatic. At this stage routine clinical examination was normal, including simple bimanual tasks such as in-phase and out-of-phase hand tapping with vision occluded (Stephan et al., 1999). However, the patient showed some subtle left-sided abnormalities when performing out-of-phase sequential hand movements on Luria’s bimanual hand co-ordination task, also with vision occluded (Luria, 1966). Follow-up imaging 4 years after diagnosis has failed to show any evidence of recurrence.

Ten right-handed normal subjects with comparable performance on simple reaction task measures were used as controls. The study was approved by the local ethics committee and all subjects gave informed, written consent.

### Imaging

**Fig. 1:**
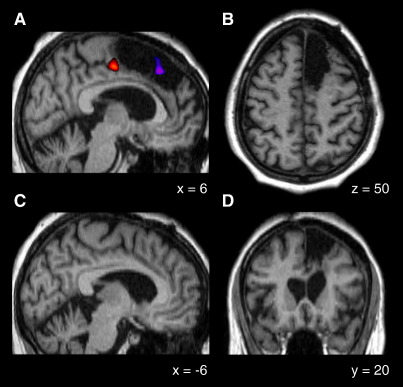
Lesion localisation. (A) Activation in the region of the SEF – a marker for the rostral extent of the SMA – superimposed on T1-weighted anatomical scan normalised in MNI space (orange). The purple area identifies the location of the rostral pre-SMA region which we have previously shown to be activated by changing oculomotor plans during conflict. (B–D) Sagittal, axial and coronal anatomical slices showing the extent of the lesion.

The lesion was manually traced on what was an otherwise normal T1-weighted 1 × 1 × 2 mm MPRAGE sequence at 1 × 1 × 2 mm resolution obtained 22 months after surgery on a 1.5 T Siemens Magnetom Vision system (see Fig. 1). The brain volume image was then transformed to the standard Montreal Neurological Institute T1 template using SPM2 (http://www.fil.ion.ac.uk/spm) non-linear coregistration routines. So as to avoid lesion-induced distortion, the normalisation parameters were derived only from normal brain regions (Brett et al., 2001).

The border between the pre-SMA and the SMA proper has been found to correspond reasonably well to the VCA line (Zilles et al., 1996); however, there is considerable inter-individual variation to which local sulcal patterns offer no reliable clues (Picard and Strick, 1996), making it hard to differentiate between these two areas with conventional imaging (Behrens et al., 2006). We wanted to establish whether the area of the SMA representing the hand was intact. Since manual movements can cause pre-SMA and SEF activation (the latter as a result of actual or suppressed gaze shifts), instead of imaging hand movements we used eye movements to localise the SEF which is rostral to the hand area of the somatopically organised SMA (Grosbras et al., 1999; Yamamoto et al., 2004). We did this by running a functional series of images contrasting blocks of saccades performed in the dark with blocks of rest (see Fig. 1A, orange). These data were collected with a T2*-weighted echo-planar sequence (TR = 4.3 s, 40 axial slices, resolution 3.5 × 3.5 × 3.5 mm) in one session of 85 volumes. The first 5 volumes were discarded to allow for magnetic saturation effects. For the remainder of the run, the patient was aurally cued to perform free saccades in the dark or to rest in alternating blocks lasting 10 volumes each. Using SPM2, the functional images were realigned, normalised to the same stereotactic space as the T1 image, and smoothed with a Gaussian kernel of 7 mm full-width at half-maximum. The data were high-pass-filtered (0.0078 Hz cutoff) to remove low-frequency signal drifts. To test for eye-movement-related activation the data were entered into a voxel-wise general linear model that included the experimental conditions, and head movement parameters derived from the realignment as effects of no interest. An appropriately weighted linear *t* contrast with a threshold of *p* < 0.05, corrected for multiple comparisons across the brain, was used to identify voxels in the region of the medial frontal wall corresponding to the patient’s SEF. Eye movements were monitored using an ASL model 504 LRO infrared video-based eye tracker (Applied Science Laboratories, Bedford, MA) sampling at 240 Hz.

The co-registered statistical parametric map derived from this series was superimposed on the anatomical scan to localise the SEF in relation to the lesion. Since the images are in normalised stereotactic space we can also demonstrate that the region of the pre-SMA that we have previously shown to be activated by conflict between motor plans (Nachev et al., 2005) falls well within the lesion (see Fig. 1A, purple).

### Psychophysics

**Fig. 2:**
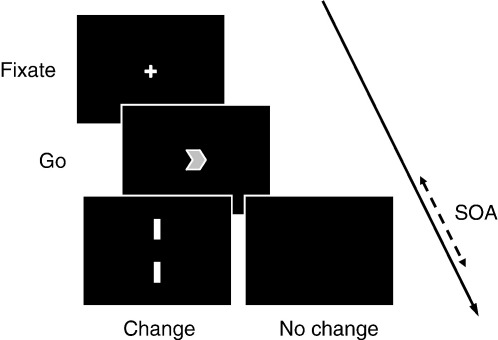
Schematic of the change-of-plan paradigm. Stimuli are not drawn to scale.

All subjects viewed a computer screen placed 60 cm in front of them and responded with either forefinger on a button box. They performed 800 pseudorandomly ordered trials of a change-of-plan paradigm in one session, consisting of 8 blocks (see Fig. 2).

Each trial began with a central fixation cross presented for 500–800 ms followed by a green arrow (go cue) of 200-ms duration which instructed the subject to make a speeded response with the corresponding hand. On 20% of trials, the green arrow was accompanied or succeeded by a pair of white bars (change cue), appearing above and below it and also lasting 200 ms, which instructed the subject to cancel the planned movement and execute a response with the opposite hand. The interval between the two cues (SOA) was automatically adjusted, depending on performance, by a staircase algorithm that increased it by 50 ms after every successful change trial, and reduced it by the same amount after every failed change trial. So as to eliminate the confounding effects of slowing following change trials, such trials were always succeeded by a go trial which was removed from the final analysis. Subjects were asked not to correct their errors but were not penalised for doing so. On the remaining 80% of trials, subjects simply executed the movement indicated by the go cue (no change trials). All stimuli were controlled using Presentation software (http://nbs.neuro-bs.com/) running with real-time priority under Microsoft Windows 98SE on a Toshiba SatPro 6000 laptop computer.

On a separate occasion, AG was tested on a manual ‘countermanding’ (or ‘stop-signal’) paradigm. She performed 900 trials exactly as in the change-of-plan task except that she was asked to withhold *any* response when she saw the change cue. Thus, in this task, control can be evaluated in the absence of competition from contralateral responses.

The measure of interest was the relation between the SOA and the probability of successfully withholding the first response. The staircase adjustment of the SOA ensured that we could counteract any strategic slowing and thereby accurately evaluate subjects’ inhibition performance. To characterise the psychometric functions we used Bayesian methods in conjunction with Markov chain Monte Carlo (MCMC) sampling (Neal, 1993). These methods have been shown to provide more accurate estimates of the parameters than analogous frequentist approaches (Kuss et al., 2005), especially when the number of observations is small as is necessarily the case in psychophysical studies involving patients. The psychometric functions were parameterised as logistic functions and maximum *a posteriori* (MAP) estimates were derived as follows. For each psychometric function, a binomial mixture model describing the threshold, slope, and lapse rate was generated using the following very broad prior distributions (parameters in parentheses): lapse rate: beta (2,50), threshold: normal (200,200), slope lognormal (4,1). The same prior was used for all functions. MCMC sampling was used to derive 5000 samples from the posterior of which the last 4800 were used to generate the MAP estimate and approximate Bayesian confidence intervals for the threshold.

## Results

Functional localisation of the SEF (coordinates 6, − 15, 52, *t* = 8.00, *p* < 0.001) demonstrated the posterior extent of the lesion to be just anterior to the SMA/pre-SMA border. Thus the lesion spared the SMA.

**Fig. 3:**
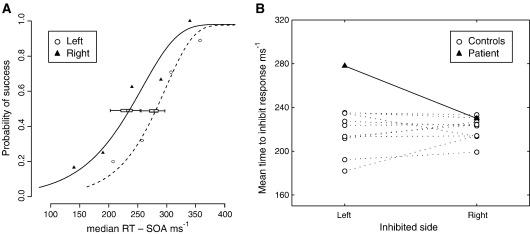
Behavioural data. (A) Psychometric inhibition functions for patient AG derived using the change-of-plan paradigm. The line plots show maximum a posteriori (MAP) estimates for the relation between the probability of inhibiting the first movement and the SOA adjusted by the median RT (effectively the time available to inhibit a response) for the left (solid line) and the right (dotted line) hands. Scatter plots of the raw data (left, white circles; right, black triangles) are also shown. The SOA values were determined by a staircase tracking algorithm that targeted the 50% performance level and therefore the sampling was slightly different between the two sides, and weighted towards the middle of the function. Only SOA values for which at least 5 data points were available were used. The SOA values were: left, 100 ms (*n* = 9), 150 ms (*n* = 24), 200 ms (*n* = 25), 250 ms (*n* = 10); right, 100 ms (*n* = 6), 150 ms (*n* = 15), 200 ms (*n* = 24), 250 ms (*n* = 20), 300 ms (*n* = 6). So as to generate functions describing the relation between the probability of changing response and the effective time available to do so, the SOAs were adjusted by subtraction from the median RT on Go trials: left = 458 ms, right = 440 ms. The horizontal box plots show 0.05, 0.25, 0.5, 0.75, and 0.95 confidence boundaries. The two functions are significantly different (Bayesian *p* = 0.002). (B) Scatterplot of the left and right hand inhibition function 50% thresholds for AG (filled markers) and the ten control subjects (unfilled markers), derived as described in A. AG differed from controls only on the left (two-sample *t* test, *p* = 0.011), and none of the controls showed a significant difference between left and right based on Bayesian MAP estimates.

On the change-of-plan task, AG’s performance revealed a marked deficit in her ability to inhibit a response of the left hand specifically on change trials, i.e. when she was instructed to change her ongoing plan to make a left hand button-press and instead instructed to move her right hand (see Fig. 3A). Inhibitory performance here is best described by the probability of inhibiting the first response on change trials as a function of the SOA adjusted for the median reaction time. MCMC estimates of the threshold (50% performance) of this function were significantly higher for the left compared with right hand (278 ms vs. 230 ms, Bayesian *p* = 0.002 based on MCMC sampling), a finding that was not observed in any of the controls. Furthermore these values differed from the controls only for the left hand (two-sample *t* test, left *p* = 0.011, right *p* = 0.39, see Fig. 3B).

By contrast, the threshold asymmetry in the pure countermanding condition (in which either one or no responses were made) was much less pronounced (216 ms for left hand vs. 198 ms, for the right, Bayesian *p* = 0.053 based on MCMC sampling), demonstrating that the lateralised deficit observed in AG was sensitive to the presence of competition between two alternative responses, i.e. as in the change-of-plan task.

Critically, this difference between the hands was not present on simple go reaction times (median values: left = 458 ms, right = 440 ms, two-sample Kolmogorov–Smirnov test asymptotic *p* = 0.22). These reaction times did not differ significantly from controls on either side (two-sample *t* test, left *p* = 0.16, right *p* = 0.18), suggesting the deficit is specific to situations where competition between actions occurs.

## Discussion

This study provides the first evidence that the human pre-SMA may be *necessary* for inhibiting competing single motor plans in situations of response conflict. It is a direct test of a previous study which found activation in a medial frontal area well within the borders of this patient’s lesion in response to conflict generated by the same paradigm, but in the oculomotor domain (Nachev et al., 2005). These results cannot be explained by damage to the SMA proper as functional imaging demonstrated that this patient’s lesion must be rostral to it.^1^ Although involvement of other anterior motor areas in the periphery of the lesion, particularly the dorsal cingulate, is possible, medial activation anterior to the VCA line in our imaging version of this task was confined to the pre-SMA (Nachev et al., 2005). Moreover, no adequately controlled lesion study to date has convincingly associated damage to the cingulate with deficits interpretable as a failure of control over immediate action (Baird et al., 2006; Fellows and Farah, 2005; Kennerley et al., 2006).

The pre-SMA is consistently activated in paradigms in which conflict is explicitly induced by requiring subjects to inhibit an established response rule in favour of another (Garavan et al., 2003; Miller et al., 2005; Nachev et al., 2005; Rushworth et al., 2002; Ullsperger and von Cramon, 2001; Wittfoth et al., 2006; Wolbers et al., 2006). Although such paradigms have sometimes used sequences, similar activation profiles are generated with single responses. Indeed, the most elemental paradigm – a race between two incompatible actions is sufficient to produce robust pre-SMA activity (Garavan et al., 2003; Nachev et al., 2005; Ullsperger and von Cramon, 2001), which is not even dependent on the subjects’ having any conscious awareness of conflict (Wolbers et al., 2006).

It may be objected that pre-SMA activity occurs too early in relation to active movement to account for the kind of inhibitory process we describe (Alexander and Crutcher, 1990; Matsuzaka et al., 1992). However, all that is required is that the timing of activity should be within the period when competing condition–action associations are co-activated. This need not be at the execution stage, indeed it would seem inefficient for resolution to occur so late. In any case, there is direct evidence of pre-SMA involvement *during* the preparation or execution of action (Isoda, 2005; Luppino et al., 1991; Nakamura et al., 1998). For example, it has been shown that when monkeys are taught to perform a simple delayed saccadic task, microstimulation of the pre-SMA in the pre-saccadic interval modulates saccadic latency in a context-dependent way (Isoda, 2005). Interestingly, when microstimulation was applied in close proximity to the go signal saccade initiation was globally delayed, whereas microstimulation earlier in the delay period led to *shorter* latency saccades for contraversive targets.

This is the kind of activity we would expect if the pre-SMA is suppressing competing plans. But even more directly, it has been shown that interfering with the function of the pre-SMA specifically alters switching from one condition–action association to another (Nakamura et al., 1999; Rushworth et al., 2002; Shima and Tanji, 1998). Furthermore, a recent report has shown remarkable single cell activity in the pre-SMA that is sensitive to switches between condition–action associations in a saccadic task switch paradigm (Isoda and Hikosaka, 2007). Importantly, the time point of differentiation in neuronal activity between switch and non-switch trials preceded the behavioural differentiation time by long enough to allow this activity to affect behaviour. Indeed, microstimulation following the cue to move resulted in significantly *improved* switching behaviour, with a conduction time well within the available time window.^2^

Moving beyond the specific evidence, we would suggest that the results of studies not directly focussed on competition between responses can also be reconciled with the unitary function we propose for the pre-SMA. As we outline below, it seems to us that our proposal is the most parsimonious way of explaining the large corpus of data on this region.

### Motor paradigms

The most general observation about pre-SMA activity in relation to overt action is that it tends to favour complex acts over simple ones (Picard and Strick, 1996). A finer and largely uncontroversial classification is based on its modulation by three contrasts: internally vs. externally guided, novel vs. well-learned, and sequential vs. single. Here we show that the conflict-related activity outlined in the Introduction can account for *all* of these contrasts without the need to invoke any higher order process.

#### Internally vs. externally guided action

The earliest studies of voluntary action noted that when a choice between two movements is not guided by an explicit cue in the immediate external environment, pre-SMA activity is greater than when such a cue is present (Deiber et al., 1991; Frith et al., 1991). Indeed, the contrast appeared to be strongest when subjects were not given any basis on which to choose one response over the other and were instructed to respond “freely”. It has been tacitly assumed that because the number of responses between the two conditions is equated the difference in brain activation must reflect a difference in the pathways engaged by internally and externally triggered movements.

In fact, these conditions are very poorly equated, and no such conclusion can be safely drawn. When subjects respond “freely” the circumstances that determine their responses are not simpler than when they are cued, but much more complicated, since removing an external criterion for responding does not make the response criterionless but merely hides the criterion from view. Critically, the range of criteria that might determine the subject’s response – whether consciously or not – is indefinite and is likely to be substantially larger than in the explicitly directed condition because it is no longer constrained by attention to a specific set of stimuli. Thus the number of condition–action associations that are automatically activated will be much greater, leading to greater need for conflict resolution, as outlined in the Introduction. In addition, this need for inhibition will be further amplified by the absence of bias in favour of any one action (Botvinick et al., 2001).

For these reasons, the apparent specialisation for internally guided action is much more simply explained by the same process of inhibiting competing motor plans being manifest to a different degree in response to differing demands. We have attempted to disprove this argument in an experiment where response “freedom” and response conflict were independently manipulated in the oculomotor domain (Nachev et al., 2005). Consistent with the hypothesis advanced here, the pre-SMA was activated by both manipulations; however, the modulation of activity was dissociable, with two close but distinct parts of the pre-SMA being responsive to either factor but not significantly to both. It is therefore conceivable that different processes were engaged by the two factors, but it is also possible that different regions within the pre-SMA were differentially engaged by the same process. Only the finding of an interaction between the two factors in the same brain region – which was not present–could be adduced as strong evidence of unitary function.

A recent sophisticated study of intention does not settle the point either (Lau et al., 2004). Adapting Libet’s paradigm (Libet et al., 1983), subjects made freely timed movements and were asked to attend to the correspondence between an external clock and, on different trials in blocks, either the perceived time of the desire to move or the time of the movement itself. The contrast between the two revealed activation in the pre-SMA, from which the authors concluded that they had identified a “representation of intention” in the human brain. It may be objected, however, that merely because subjects attended to the time when they believe they felt a desire to move does not imply that the areas activated in their brains represented their intention.

First, being conscious of a desire to move is neither a necessary nor a sufficient criterion for voluntary action (Bennett and Hacker, 2003). One would not think of oneself as having moved involuntarily if one were not aware of a conscious desire to move just before the movement itself, and, equally, one would not call a movement voluntary solely because it was preceded by an urge to move (or else sneezing would be a voluntary act). What the subjects were attending to therefore cannot be unambiguously related to anything of consequence to voluntary action. Secondly, attending to one’s intention – however conceptualised – need not necessarily activate a brain region concerned with intention, any more than attending to one’s anger need identify a region representing anger. A much simpler explanation of the data in this experiment is that subjects attended more carefully to their actions in the blocks where they had to attend to the timing of the “desire to move”, thereby leading to activation of a wider range of condition–action associations and consequently resulting in greater pre-SMA activity as previously described.

#### Novel vs. well-learned action

Pre-SMA activity has been shown to be greater when novel actions are compared with well-rehearsed ones (Hikosaka et al., 1996; Kawashima et al., 1998; Nakamura et al., 1998), and to decrease during motor learning (Sakai et al., 1999). Disruption of the pre-SMA has been shown to interfere with the acquisition of new sequences but possibly not established ones (Nakamura et al., 1999). On this basis it has been proposed that the pre-SMA is critically involved in establishing new S–R associations (Nakamura et al., 1998; Sakai et al., 1999; Sakai et al., 1998). However, none of the extant data allows us to distinguish a critical role in such learning from the role in resolving conflict between condition–action associations we have suggested.

Consider the case where an animal has to learn, by trial and error, an appropriate motor response when confronted with a particular sensory stimulus (or external condition). Positive or negative feedback helps to establish strong condition–action associations as the animal learns the correct response for each particular stimulus (or condition). But such learning necessarily entails the inhibition of competing responses. Moreover, the degree of inhibition will tend to covary tightly with learning. A poorly learned association will inevitably generate greater competition between contingent responses than a well-learned one because there will *necessarily* be less bias in its favour. But as the association becomes better established, the increased bias towards it will reduce the need for inhibition of competing plans.

Finding a condition–action-specific pattern of activation would not be decisive in making the distinction between learning vs. resolving competition between motor responses, since the pattern of inhibition may well differ depending on the array of condition–action representations being inhibited.

One fact, however, is strongly against the learning account: activity in the *same* learning-related cells reappears during switching from one well-learned action to another (Nakamura et al., 1998), which is clearly surprising for a purely learning-related role but would be entirely consistent with inhibition of competing action plans. It must nevertheless be conceded that these two accounts will always be difficult to differentiate, as successful inhibition of competing alternatives will clearly be essential to rehearsal on which learning self-evidently depends.

#### Single vs. sequential action

Perhaps the most carefully explored aspect of the pre-SMA is its marked activity during sequences of movements (Hikosaka et al., 1996; Kawashima et al., 1998; Shima et al., 1996; Shima and Tanji, 1998, 2000). This has lead to the notion that the pre-SMA has a special role in movement sequences, and, by implication, that it is part of a neural circuit specialised for various aspects of sequential action. Carefully considered, this notion is implausible. Virtually all voluntary movements involve the sequential contraction of different muscle groups that do not always operate together and are therefore unlikely to have unitary representations at a cortical level. All action is therefore sequential to some degree. If the SMC had a special function in sequencing actions we would therefore expect it to show differential activation between two “simple” movements that differ in the number of independently operable muscle groups involved: e.g. a reaching hand movement vs. an eye movement. This has not been shown to be the case.

What we need to consider is the peculiar – and artificial – nature of the movement sequence paradigms used to study this phenomenon. An action is considered as a sequence of movements if it is composed of several movements that can each be – and often habitually are – made independently: e.g. a sequence of saccades. By contrast, the oculomotor pursuit of a target describing a smooth arc across the visual field is not considered a sequence even though it involves sequentially shifting changes in the contraction of the ocular muscles. Furthermore, experimental sequences usually require the subject to perform the same component movements but in a different order within the same experiment: this is obviously necessary so as to distinguish between activity due to each component from that due to their interaction as part of a sequence. Finally, the movements are considered a sequence – rather than just a series of individual trials – because they are cued at the onset of the first movement only. Thus, in these paradigms, each component movement is associated with a cue (or some other condition) and what differs across trial types is just the timing of its execution. The result is that condition–action associations are established for each component movement that initially differ only in their relative timings, if at all. At the early stages of learning, presentation of the initial cueing stimulus will therefore activate *all* condition–action associations involved to some degree, giving rise to conflict requiring appropriate inhibition. Since the variation between one sequence and another is at the time point of each component movement initiation, this is when we would expect pre-SMA activity to occur to resolve conflict between competing motor plans. Once the sequence is well learnt, the order of each component becomes part of the condition on which the response is selected and therefore there will be less requirement for conflict resolution, resulting in reduced pre-SMA activity. The pre-SMA may therefore facilitate the encoding of a sequence, but only to the extent to which it inhibits alternative responses so that the sequence may be successfully performed enough times for the encoding to take place. There is no need to suggest it *represents* sequence information for later retrieval, indeed neurons exhibiting sequence-specific activity have recently been shown to be 6 times commoner in M1 than in the SMC (Lu and Ashe, 2005).

### Non-motor paradigms

The pre-SMA has been implicated in functions that do not involve action by the subject. Since pre-SMA activity often precedes movement execution it is not surprising to find that it accompanies movements that are not executed but only imagined. Indeed, the most detailed study of single-cell activity in the human SMC found some neurons whose activity is *greater* during imagined movements than executed ones (Amador and Fried, 2004). If this region is involved in the suppression of competing motor plans, this is precisely what we would expect. It would be very surprising indeed if imagining a movement did not involve suppressing a conscious desire to execute it.

Activity during the observation of action (Rizzolatti and Luppino, 2001) can be explained on analogous lines. Since an action is usually tightly coupled to its goal – especially the type of actions used in observation experiments – it is inevitable that observing an action will activate neural circuits concerned with movements intended to secure the same or similar goals. Moreover, action observation experiments usually employ hand grasping gestures which the subject will generally observe when performing them himself, thereby linking the observation of an action to its execution *independently* of its goal.

That it is at no point the subject’s intention to execute any of these movements is irrelevant: the brain does not *know* what the subject has been instructed to do: automatic activation will therefore occur, requiring inhibition. Thus activity here need not have anything to do with the *recognition* of action, and indeed studies showing such a perceptual deficit following interference with the pre-SMA are notably absent. Nor is there any necessity to invoke a mechanism for learning from imitation, unless it can be shown that disrupting the pre-SMA during the observation of an action subsequently impairs its learning.

Evidence that the pre-SMA is *necessary* for non-motor aspects of cognitive operations is similarly sparse. Merely correlational evidence is hard to interpret given that cognitive paradigms almost invariably involve a response of some sort. Moreover, paradigms with a spatial component usually require gaze suppression, and those with a verbal component often lead to silent or suppressed verbalisation, both of which involve motor processing. Even when these things are carefully controlled for (Hanakawa et al., 2002), one would expect tasks that are cognitively more difficult to activate the pre-SMA to a greater degree because, *necessarily*, in these situations the bias towards one response compared with another is less and/or the range of condition–action associations concurrently activated is greater, both factors to which a competition resolving mechanism would be expected to be sensitive, as we have already argued.

One case needs special consideration: the widespread belief that the pre-SMA is involved in temporal discrimination. The evidence for this rests almost entirely on functional imaging studies that have shown activation in this area when making a temporal judgment is contrasted with making some other kind of judgment (Coull et al., 2004; Pastor et al., 2006). The problem is that these two conditions are never sufficiently equated in terms of the relative propensity to activate condition–action associations.

Just as imagining or observing an action may be expected automatically to activate neural substrates concerned with action, so a context more readily linked to action will result in greater activity in these areas compared with one less so. At the time scales explored in such experiments, by far the commonest reason for an object to change in the temporal dimension is because it is moving. All movement, like all action, *necessarily* takes place over time, which is not true of other properties of objects such as colour, shape or size. For this reason, we would argue, attending to the temporal aspect of a stimulus is much more likely to activate neural circuits concerned with action than attending to some other aspect of it – even if no motor act takes place.

An alternative interpretation of the activation of *any* motor area during such tasks is therefore the automatic activation of condition–action associations, and their consequent inhibition. Showing that these effects are modulated by voluntary attention does not overcome this criticism, as both motor and sensory unconscious processes are sensitive to attentional manipulations (Montaser-Kouhsari and Rajimehr, 2005; Naccache et al., 2002; Sumner et al., 2006). Moreover, there is both behavioural (Anderson et al., 2002; Tucker and Ellis, 1998, 2004) and neurophysiological (Grezes and Decety, 2002) evidence of motor activation in a context more subtly related to action – the purely passive viewing of figures with affordances – so the process we are describing here is far from hypothetical. Even highly abstract figures with minimally lateralised affordances can produce appropriately lateralised motor cortex activation.^3^

Naturally, we are not asserting that these observations prove that the pre-SMA is not involved in temporal discrimination or any other perceptual task, but merely that *the case has not been made.* If we are to believe that an area concerned with actions – functionally and anatomically – is also involved in a highly specific aspect of perception, we need better evidence than functional imaging, and rather closer attention to confounds that may point to alternative explanations that are both simpler and more easily reconciled with the extant literature.

## Conclusion

We have demonstrated that pre-SMA injury can lead to a selective deficit in the ability to inhibit a response in the context of competition between actions. These findings reflect a cardinal role for this region in the process of resolving competition between action contingencies so that coherent behaviour may emerge. We propose that this process *alone* may explain the vast bulk of extant data on this region, and that there is therefore no need to invoke less elemental functions in our description of the role of the pre-SMA in action control.

Such merit as the framework proposed here has rests on its being the most economical explanation of the facts. Naturally, the brain need not be parsimonious in its organisation, and therefore direct evidence of unitary function is required. One way of achieving this with functional imaging is to explore paradigms where two putatively dissociable processes are manipulated in a factorial manner: the presence of an interaction between the factors would suggest that they are not independent, although of course the absence would not preclude it. Alternatively, and perhaps more powerfully, imaging measures of neuronal adaptation (Grill-Spector and Malach, 2001) could be used to test if neurons in the same location are dissociably activated by two potentially different processes, a technique that has shown some success in the sensory domain. In any event, it is essential that hypotheses generated based on functional imaging are subsequently tested with functional interference.

More generally, we would suggest that the proliferation in functions attributed to this region should be viewed critically, and greater efforts should be made to integrate the evidence across the field, as we have attempted to do here.

## Footnotes

1 In line with the peculiarly polarised pattern of dysfunction seen after damage to large regions with medial frontal cortex (Laplane et al., 1977) – either absence of spontaneous movement or disinhibited semi-purposeful movement – it is conceivable that failure of inhibition following damage to the pre-SMA requires an intact SMA in order to become manifest, as the pre-SMA may act by inhibiting the SMA in the normal state.

2 It should be noted that the inhibitory deficit seen in our patient is in the context of competition between alternative movements rather than between a movement and no movement at all. This raises the possibility that the inhibitory failure is secondary to a failure adequately to activate the alternative response which is normally inhibitory of any other responses. However, it would be odd for the principal deficit here to involve processes plausibly dominated by the contralesional hemisphere.

3 S. J. Anderson, personal communication.
